# Oral pre‐exposure prophylaxis preference, uptake, adherence and continuation among adolescent girls and young women in Kampala, Uganda: a prospective cohort study

**DOI:** 10.1002/jia2.25909

**Published:** 2022-05-11

**Authors:** Yunia Mayanja, Onesmus Kamacooko, Jane Frances Lunkuse, Vincent Muturi‐Kioi, Allan Buzibye, Denis Omali, Kundai Chinyenze, Monica Kuteesa, Pontiano Kaleebu, Matt A. Price

**Affiliations:** ^1^ Medical Research Council/Uganda Virus Research Institute and London School of Hygiene and Tropical Medicine (MRC/UVRI & LSHTM), Uganda Research Unit Entebbe Uganda; ^2^ IAVI Nairobi Kenya; ^3^ Infectious Diseases Institute College of Health Sciences Makerere University Kampala Uganda; ^4^ IAVI New York New York USA; ^5^ Department of Epidemiology and Biostatistics University of California San Francisco San Francisco California USA

**Keywords:** adolescent girls and young women, sub‐Saharan Africa, HIV prevention, pre‐exposure prophylaxis, adherence, retention

## Abstract

**Introduction:**

Oral pre‐exposure prophylaxis (PrEP) has been scaled up; however, data from real‐world settings are limited. We studied oral PrEP preference, uptake, adherence and continuation among adolescent girls and young women (AGYW) vulnerable to HIV in sub‐Saharan Africa.

**Methods:**

We conducted a prospective cohort study among 14‐ to 24‐year‐old AGYW without HIV who were followed for 12 months in Kampala, Uganda. Within at least 14 days of enrolment, they received two education sessions, including demonstrations on five biomedical interventions that are; available (oral PrEP), will be available soon (long‐acting injectable PrEP and anti‐retroviral vaginal ring) and in development (PrEP implant and HIV vaccine). Information included mode and frequency of delivery, potential side effects and method availability. Volunteers ranked interventions, 1 = most preferred to 5 = least preferred. Oral PrEP was “preferred” if ranked among the top two choices. All were offered oral PrEP, and determinants of uptake assessed using Poisson regression with robust error variance. Adherence was assessed using plasma tenofovir levels and self‐reports.

**Results:**

Between January and October 2019, 532 volunteers were screened; 285 enrolled of whom 265 received two education sessions. Mean age was 20 years (SD±2.2), 92.8% reported paid sex, 20.4% reported ≥10 sexual partners in the past 3 months, 38.5% used hormonal contraceptives, 26.9% had chlamydia, gonorrhoea and/or active syphilis. Of 265 volunteers, 47.6% preferred oral PrEP. Willingness to take PrEP was 90.2%; however, uptake was 30.6% (*n* = 81). Following enrolment, 51.9% started PrEP on day 14 (same day PrEP offered), 20.9% within 30 days and 27.2% after 30 days. PrEP uptake was associated with more sexual partners in the past 3 months: 2–9 partners (aRR = 2.36, 95% CI: 1.20–4.63) and ≥10 partners (aRR 4.70, 95% CI 2.41–9.17); oral PrEP preference (aRR 1.53, 95% CI 1.08–2.19) and being separated (aRR 1.55, 95% CI 1.04–2.33). Of 100 samples from 49 volunteers during follow up, 19 had quantifiable tenofovir levels (>10 μg/L) of which only three were protective (>40 μg/L).

**Conclusions:**

Half of AGYW preferred oral PrEP, uptake and adherence were low, uptake was associated with sexual behavioural risk and oral PrEP preference. Development of alternative biomedical products should be expedited to meet end‐user preferences and, community delivery promoted during restricted movement.

## 1 INTRODUCTION

During the period 2010–2020, new HIV transmissions reduced by 30% globally. Despite this, the 2020 global target of fewer than 500,000 new HIV diagnoses annually was not achieved, and sub‐Saharan Africa (SSA) contributed 54.7% to new infections in 2020 [1, 2]. No single HIV prevention intervention has 100% uptake and adherence, therefore, combination prevention that includes behavioural, biomedical and structural approaches is key. In 2018, the World Health Organization developed specific guidelines recommending the use of daily oral tenofovir‐based pre‐exposure prophylaxis (PrEP) as part of combination prevention for adolescent girls and young women (AGYW) behaviourally vulnerable to HIV [3]. Mathematical models indicate that incidence will reduce substantially if 25% of HIV investments go to effective combination prevention [4].

Oral PrEP is one of the biomedical interventions available to AGYW who account for 25% of new HIV diagnoses in SSA [1]. AGYW are vulnerable biologically due to an immature cervix, genital mucosal disintegration due to sexually transmitted infections (STIs), intravaginal practices and forced sex [5, 6, 7]. Further vulnerabilities are posed by inadequate schooling, food insecurity, discriminatory cultural norms, intimate partner violence (IPV) and age‐disparate and/or transactional relationships [8]. Oral PrEP, therefore, provides a user‐controlled method, where condom negotiation is limited. PrEP implementation projects funded through the US President's Emergency Plan for AIDS Relief (PEPFAR) [8] indicate that uptake has been low and slow [9]. Reports from family planning clinics in Kenya and the Sustainable East Africa Research for Community Health (SEARCH) study showed low uptake among individuals <25 years [10, 11]. Conversely, the Prevention Options for Women Evaluation Research (POWER) project among AGYW in Kenya and South Africa reported high uptake (90%) associated with behavioural risk, IPV, depression and STIs, but also early discontinuation due to side effects, challenges with access and daily dosing [12].

Product attributes are a concern among AGYW who emphasize discrete packaging for end‐user privacy [13]. Indeed, ending HIV hinges not only on providing prevention interventions but also options that meet end‐user needs and preferences [4]. Data on real‐world PrEP uptake, complemented by tenofovir adherence assessments among AGYW in SSA, are still limited. In this study of 14‐ to 24‐year‐old Ugandan AGYW who frequently reported paid sex, we evaluated preference, uptake, adherence and continuation on oral PrEP in order to provide comprehensive data on these indicators and suitability for this population.

## 2 METHODS

### 2.1 Study design

We conducted a prospective cohort study (January 2019–December 2020) at the Good Health for Women Project (GHWP) clinic, which offered daily oral PrEP to AGYW.

### 2.2 Setting

The GHWP clinic was established in Kampala, Uganda, in 2008 and until December 2020, provided HIV prevention and treatment, and sexual reproductive health services to women behaviourally vulnerable to HIV, and conducted HIV vaccine preparedness studies.

### 2.3 Sampling, recruitment and study procedures

Field workers recruited peer leaders with whom they mobilized volunteers from 22 communities in southern (10) and northern (12) Kampala located within the site's catchment area. These were mainly urban slums characterized by entertainment facilities, where sex work, alcohol and illicit drug use were common. Volunteers were pre‐screened to ascertain that minors (14–17 years) were emancipated/mature minors, who could legally consent to participate [14]. From January to October 2019, we consecutively enrolled volunteers who were sexually active in the 3 months before enrolment; aged 14–24; HIV negative; and willing to undergo study procedures. All were PrEP naïve and each volunteer followed for 12 months. We screened for hepatitis B to avoid the risk of worsening infection if the chronically infected started and stopped PrEP. We hypothesized that PrEP uptake would be high (up to 80%) among AGYW.

#### 2.3.1 Screening

Trained research nurses gave volunteers study information, answered their questions and administered an assessment of understanding (AoU); those who answered 8 of 10 questions with two attempts were consented and study screening done. Assessment for being at substantial risk for HIV infection was done using the national risk screening tool provided by the Ministry of Health. The tool assessed for risk in the past 6 months: (1) being sexually active *PLUS* one of the following: condomless vaginal or anal sexual intercourse with >1 partner, a sexual partner with ≥1 risk (injects drugs, has sex with men, transgender, sex worker and has condomless sex with multiple partners), STIs and use of post‐exposure prophylaxis; (2) sharing injections and (3) having an HIV‐positive sexual partner not on effective anti‐retroviral (ARV) therapy. PrEP eligibility was further ascertained by having *ALL* the following: HIV‐negative test, responding *(yes)* to any of the substantial risk criteria described above, hepatitis B negative and normal creatinine clearance.

Volunteers were assessed for the awareness of novel biomedical HIV interventions, given basic oral PrEP education, and assessed for willingness to take daily oral PrEP. An appointment for possible enrolment was scheduled within 2 weeks.

#### 2.3.2 Enrolment

Research nurses gave volunteers the first education session on five biomedical HIV prevention interventions which are: available (oral PrEP), will be available soon (long‐acting (LA) injectable PrEP and ARV vaginal ring) and in development (PrEP implants and HIV vaccines). Nurses used a tool developed by the study team, translated into Luganda (the local dialect) and approved by the Ethics Committee. Discussions included mode and frequency of method delivery, potential side effects, method availability, demonstration of available samples (oral PrEP and ARV vaginal ring) and use of licensed vaccines or contraceptive proxies for other methods. The next visit was scheduled 14 days later; volunteers received a similar second education session and gave their preference by ranking the five methods on a 1 to 5 scale (1 = “most preferred,” 5 = “least preferred”). Willing volunteers received 1 month's supply of tenofovir disoproxil fumarate/lamivudine (TDF/3TC) and an adherence card with instructions to mark immediately after a pill was taken, and leave the day's slot blank if a pill was not taken. Instructions on the card were both in English and Luganda. The study staff went through the card with the volunteer to ensure they understood how to use it. This formed the basis for self‐reported adherence, which was complemented by pill counts, that is the number of days marked on the card would be the same as number of pills taken per month. A sample adherence card is included.

#### 2.3.3 Follow up

From enrolment, visits were conducted after every 3 months. Volunteers received HIV risk reduction counselling, pregnancy and STI testing and contraceptive re‐fills. Those on PrEP had monthly visits for self‐reported adherence and HIV risk assessments, and quarterly blood draws for TDF assessments. The field worker called volunteers whose scheduled visits were due. They were reimbursed $5.6 per visit. Home visits were done for volunteers who missed study visits.

### 2.4 Data collection

We collected data using interviewer administered questionnaires. Screening data included: HIV risk, willingness to take PrEP and sample test results, that is HIV, syphilis, hepatitis B, pregnancy and creatinine. Creatinine clearance (Cockcroft–Gault) was used to determine the safety of PrEP uptake. Enrolment data included product preference and STIs. Self‐reported adherence data were collected monthly, while TDF samples were scheduled at quarterly study visits, and stored at –80°C for batch testing.

Laboratory technologists tested serum for HIV: Determine (screening), Statpak (confirmatory), SD Bioline (tiebreaker), hepatitis B (Roche Cobas e 411 assay, Germany), creatinine (Roche diagnostics Creatinine plus version 2.0 (CREP2)) and syphilis (Rapid Plasma Reagin/Treponema Pallidum Particle Agglutination Assay). Plasma TDF was quantified by Thermo Scientific LCQ Fleet ion trap liquid chromatography–mass spectrometry (LC/MS^n^) model operated by Xcalibur™ software. Endo‐cervical swabs were tested (chlamydia/gonorrhoea) using GeneXpert (Cepheid AB, Rontgenvagen 5, Soina Sweden).

### 2.5 Study variables

Primary outcome(s)

- Oral PrEP preference; volunteer ranked preference of five interventions 1 = “most preferred” to 5 = “least preferred” and further categorized into a binary outcome with oral PrEP “preferred” if scored 1 or 2.
- Oral PrEP uptake; (Yes/No) defined as a volunteer accepting the offer of oral PrEP and picking the first 1‐month supply.

Secondary outcomes

(iii) Retention on PrEP; measured in months from the date of PrEP uptake to the date of completion of the last PrEP supply.
(iv) Adherence was measured using:
  - Plasma TDF levels at two concentration thresholds; 40 μg/L consistent with daily dosing and protective [15], and 10 μg/L the lower limit of quantification, consistent with less than daily dosing. Results were categorized as: ≥40 μg/L (quantifiable, protective), ≥10 μg/L (quantifiable, not protective) and <10 μg/L (unquantifiable).
  - Self‐reported adherence measured as the total number of pills taken since the last PrEP refill divided by the number of days since the last PrEP refill. Adherence was categorized into optimal (≥90%) and sub‐optimal (<90%).

Independent variables were assessed at (1) enrolment: current age, marital status, highest education level, main job, willingness to take oral PrEP, alcohol use (AUDIT [Alcohol Use Disorder Identification Tool]) categorized by AUDIT score as low risk (0–7); moderate risk (8–15) and high risk (16–40), and (2) enrolment and follow up: number of sexual partners in the past 3 months and knowledge of partner HIV status. Binary (Yes/No) variables assessed for the past 3 months were: condom use with sexual partners, having STIs and frequent travel from home (away from home ≥3 days a week).

### 2.6 Data analysis

Data were double entered into Open Clinica, cleaned and exported to STATA 15.0 (StataCorp, College Station, TX, USA) for analysis. Using chi‐squared tests, we compared differences between volunteers who completed and those who did not complete the study by looking at age, marital status, main job, education level, number of sexual partners, condom use with sexual partners, frequent travel from home, knowledge of partner HIV status and alcohol use. We considered variables from the literature and fitted a Poisson model with robust error variance to assess determinants of uptake. Only age (selected a priori) and factors for which the unadjusted association attained statistical significance at the *p*≤0.15 level using a likelihood ratio test (LRT) were considered for the multivariable model. Furthermore, variables were retained only if independently associated with PrEP uptake (*p*<0.15) after adjusting for the other variables. This resulted in some core variables being independently associated with PrEP uptake. We further assessed the best model by using the Akaike Information Criterion. Age was maintained in the model regardless of its significance.

### 2.7 Ethical considerations

The study protocol included enrolment of emancipated/mature minors as defined in the national guidelines [14]. The Uganda National Council for Science and Technology (HS 2435) and Uganda Virus Research Institute‐Research Ethics Committee (GC/127/18/06/658) approved the study. We obtained written informed consent from all volunteers.

## 3 RESULTS

### 3.1 Screening profile of study volunteers

We approached 561 volunteers; 29 were not screened due to: language barrier (9), not emancipated/mature minor (7), failed AoU twice (5) and other reasons (8). Therefore, 532 were screened, 154 screened out as follows: not sexually active in the past 3 months (51); HIV positive (29); hepatitis B infection (10); unwilling to undergo study procedures (8); and other reasons (56). Ninety‐three eligible volunteers were not enrolled because the sample size was achieved. We enrolled 285 AGYW of whom 265 (93.0%) completed the two education sessions on biomedical interventions and were offered oral PrEP.

### 3.2 Baseline characteristics and awareness of biomedical interventions

Volunteer mean age was 20 years (SD±2.2), 12.1% were aged ≤17 years, 55.1% had attained secondary education or higher and 21.9% reported sex work as their main occupation. Volunteers reported: ≥10 sexual partners (20.4%) and transactional sex (92.8%) in the past 3 months. Hormonal contraceptives (mainly implants and injectable) were used by 38.5% and STI prevalence (chlamydia, gonorrhoea and/or active syphilis) was 26.9%. Baseline awareness of interventions was: oral PrEP (24.5%), LA injectable PrEP (4.2%), ARV vaginal ring (2.3%) and HIV vaccine (1.5%). None knew about the ARV implant. After receiving brief information and pill demonstration, 90.2% expressed willingness to use daily oral PrEP (Table 1).

**Table 1:** Baseline characteristics of AGYW who were offered oral PrEP, Kampala (2019–2020)

Variables	Frequency (*N* = 265)	Percent (%)
Age at enrolment (years)		
14–19 (adolescents)	102	38.5
20–24 (young women)	163	61.5
Highest education level attained		
< Secondary	119	44.9
≥ Secondary	146	55.1
Marital status		
Single (never married)	152	57.3
Married	76	28.7
Separated/divorced	37	14.0
Weekly income (United States Dollar)		
≤10	171	64.5
>10	94	35.5
Main job		
No job	66	24.9
Entertainment/hospitality/other job	141	53.2
Sex work	58	21.9
Number of biological children		
None	94	35.5
≥ One	171	64.5
Alcohol use		
Low risk/abstinence	180	67.9
Moderate risk/hazardous	46	17.4
High risk (harmful and alcohol dependent)	39	14.7
Illicit drug use in the past month		
Yes	43	16.2
Frequent travel from home in the past 3 months		
Yes	110	41.5
Number of sexual partners in the past 3 months		
1	82	30.9
2–9	129	48.7
≥10	54	20.4
Condom use with sexual partners in the past 3 months		
Yes	194	73.2
Received payment for sex in the past 3 months		
Yes	246	92.8
Forced to have sex in the past 3 months		
Yes	62	23.5
Knowledge of partner HIV status		
HIV positive	5	1.9
HIV negative	45	17.0
Do not know	215	81.1
Baseline knowledge of oral pre‐exposure prophylaxis		
Yes	64	24.5
Willingness to take daily oral pre‐exposure prophylaxis		
Yes	239	90.2
Use of hormonal contraception at enrolment		
Yes	102	38.5
STIs (chlamydia/gonorrhoea/active syphilis) at enrolment^a^		
Yes	71	26.9
No	193	73.1

^a^ One volunteer not screened for sexually transmitted infections.

### 3.3 Oral PrEP preference and uptake

Of 265 volunteers, 47.6% preferred oral PrEP; 30.6% (*n* = 81) started PrEP; and 184 gave their main reason for declining as: low HIV risk perception (33.7%), dislike for daily pills (27.2%), not being ready (21.7%), preference for other methods (e.g. condoms [12.5%]), concern about side effects (2.7%) and stigma (2.2%). A higher proportion of PrEP starters reported ≥10 sexual partners versus <10 sexual partners (61.1% vs. 22.8%); *p*<0.001. Volunteers reporting ≥10 sexual partners reported 94.4% condom use versus 67.8% among those reporting <10 sexual partners (*p*<0.001).

### 3.4 Factors associated with PrEP uptake

In adjusted analysis, PrEP uptake was higher among volunteers who: reported 2–9 partners (aRR 2.36, 95% CI 1.20–4.63), reported ≥10 partners (aRR 4.70, 95% CI 2.41–9.17); preferred oral PrEP to other biomedical methods (aRR 1.53, 95% CI 1.08–2.19); and were separated (aRR 1.55, 95% CI 1.04–2.33) (Table 2).

**Table 2:** Factors associated with PrEP uptake among 265 AGYW in Kampala, Uganda (2019–2020)

Variable	Categories	*n* (row %)	Unadjusted relative risk (95% CI)	LRT *p*‐value	Adjusted relative risk (95% CI)
Overall		81 (30.6)			
Age at enrolment (years)				0.382	
	14–19	28 (27.5)	1.00		1.00
	20–24	53 (32.5)	1.18 (0.81–1.74)		1.07 (0.74–1.54)
Marital status				**0.009**	
	Single (never married)	45 (29.6)	1.00		1.00
	Married	17 (22.4)	0.76 (0.46–1.23)		0.88 (0.56–1.39)
	Separated/divorced	19 (51.4)	1.73 (1.16–2.58)		**1.55 (1.04–2.33)**
Main job				**0.002**	
	No job	16 (24.2)	1.00		–
	Entertainment/hospitality/other	36 (25.5)	1.05 (0.63–1.76)		
	Sex work	29 (50.0)	2.06 (1.25–3.40)		
Weekly income (United States Dollar)				**0.022**	
	≤10	44 (25.7)	1.00		–
	>10	37 (39.4)	1.53 (1.07–2.19)		
Number of sexual partners in the past 3 months				**<0.001**	
	1	9 (11.0)	1.00		1.00
	2–9	39 (30.2)	2.75 (1.41–5.39)		**2.36 (1.20–4.63)**
	≥10	33 (61.1)	5.57 (2.90–10.7)		**4.70 (2.41–9.17)**
Condom use with sexual partners in the past 3 months				**0.001**	
	No	11 (15.5)	1.00		
	Yes	70 (36.1)	2.33 (1.31–4.14)		
Alcohol use				**0.003**	
	Low risk/abstinence	43 (24.0)	1.00		–
	Moderate risk/hazardous	20 (43.5)	1.82 (1.19–2.77)		
	High risk	18 (46.2)	1.93 (1.26–2.97)		
Frequent travel from home in the past 3 months				**0.024**	
	No	39 (25.2)	1.00		–
	Yes	42 (38.2)	1.52 (1.06–2.18)		
Preference for oral PrEP				**0.002**	
	Not preferred (score 3, 4 or 5)	31 (22.3)	1.00		1.00
	Preferred (score 1 or 2)	50 (39.7)	1.78 (1.22–2.60)		**1.53 (1.08–219)**
Willingness to take daily oral PrEP				**0.016**	
	No	3 (11.5)	1.00		–
	Yes	78 (32.6)	2.83 (0.96–8.34)		
Had STIs (chlamydia, gonorrhoea and active syphilis)^a^				0.652	
	No	57 (29.5)	1.00		–
	Yes	23 (32.4)	1.09 (0.73–1.63)		

The bold values indicate variables that achieved statistical significance at that level of analysis.

Abbreviations: CI, confidence interval; PrEP, pre‐exposure prophylaxis; STIs, sexually transmitted infections.

^a^Excludes one volunteer who was not screened for STIs.

### 3.5 Retention of 265 AGYW who were offered PrEP (2019–2020)

**Figure 1:**
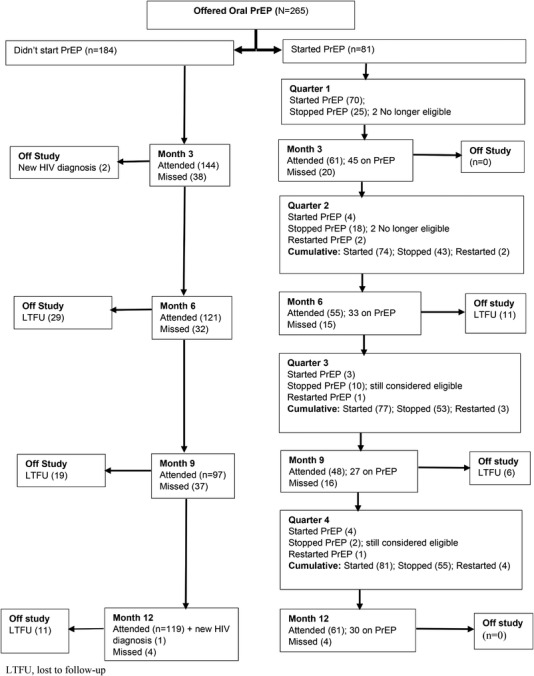
Retention of 265 AGYW who were offered PrEP (2019–2020).

Median follow‐up time on study was 5.51 months. Among PrEP starters, 51.9% started 2 weeks after enrolment (same day PrEP offered), 20.9% within 30 days and 27.2% after 30 days. The median (IQR) time on PrEP was 2.52 months. Fifty‐five volunteers discontinued PrEP as follows: lost to follow up (17), side effects (12), no longer interested or lower perceived HIV risk (9) and other reasons, for example pill burden, stigma (17). Four of these re‐started, therefore, 30 volunteers (37.0%) completed the study on PrEP. We were unable to assess if 17 lost volunteers remained eligible for PrEP; however, 34 of 38 who discontinued were still eligible. We observed (75.3%, *n* = 61) PrEP starters completed the study versus (64.7%, *n* = 119) who declined (*p* = 0.088) (Figure 1).

### 3.6 Effect of CoVID‐19

The nationwide COVID‐19 lockdown (25 March–31 May 2020) instituted 9 months before study end interrupted study visits. Month 3 and month 6 visits were 100% and 88% completed, respectively. During the lockdown, missed (*not attended*) and late (*outside schedule*) visits contributed 79.8% and 100% of month 9 and month 12 visits, respectively. We gave PrEP refills at the clinic (6) and in the community (10); three volunteers on PrEP could not be traced. After easing of the lockdown, month 9 and month 12 visits attended on schedule were 62.3% and 48.3%, respectively, but missed and late visits continued. Details are given in File S1. We did not find significant differences between volunteers who completed follow‐up and those who did not (Table 3).

**Table 3:** Comparison of volunteer characteristics between those who completed follow‐up and those who did not

Characteristic	Study completed (*n* = 180) *n* (col%)	Study not completed (*n* = 85) *n* (col%)	Chi‐square test *p*‐value
Age (years)			0.315
14–19 (adolescents)	73 (40.6)	29 (34.1)	
20–24 (young women)	107 (59.4)	56 (65.9)	
Highest education level			0.402
<Secondary	84 (46.7)	35 (41.2)	
≥Secondary	96 (53.3)	50 (58.8)	
Marital status			0.724
Married	49 (27.2)	27 (31.8)	
Separated/divorced	25 (13.9)	12 (14.1)	
Single (never married)	106 (58.9)	46 (54.1)	
Main job			0.975
No job	45 (25.0)	21 (24.7)	
Entertainment/hospitality/other	95 (52.8)	46 (54.1)	
Sex work	40 (22.2)	18 (21.2)	
Alcohol use			
Low risk/abstinence	62 (72.9)	118 (65.6)	
Moderate risk/hazardous	10 (11.8)	36 (20.0)	0.253
High risk (harmful and alcohol dependent)	13 (15.3)	26 (14.4)	
Frequent travel from home in the past 3 months			
No	102 (56.7)	53 (62.3)	0.381
Yes	78 (43.3)	32 (37.7)	
Number of sexual partners in the past 3 months			
<10	145 (80.6)	66 (77.6)	
≥10	35 (19.4)	19 (22.4)	
Condom use with sexual partners in the past 3 months			0.946
No	48 (26.7)	23 (27.1)	
Yes	132 (73.3)	62 (72.9)	
Knowledge of partner HIV status			
HIV positive	3 (1.7)	2 (2.3)	
HIV negative	32 (17.8)	13 (15.3)	0.828
Do not know	145 (80.6)	70 (82.4)	

### 3.7 PrEP adherence among AGYW

After receiving the first PrEP supply, six volunteers did not return to the clinic, 26 stopped PrEP during the quarter (between study visits) and did not provide any samples at return visits. Of 100 plasma samples available from 49 volunteers providing at least one follow‐up sample, 19.0% had quantifiable TDF. Protective TDF levels were seen in the first 3 months on PrEP among 3/42 (7.1%) volunteers despite 33 having reported optimal adherence. Additional details are given in File S2. Optimal adherence was commonly reported across follow‐up, but did not match assay data at any visit (Table 4).

**Table 4:** Self‐reported adherence versus tenofovir assay results of AGYW

Plasma tenofovir levels and self‐reported adherence	Follow‐up period of PrEP starters after study enrolment (months)			
	1–3 months	4–6 months	7–9 months	10–12 months
	*n* (%)	*n* (%)	*n* (%)	*n* (%)
Total persons on PrEP in the quarter	45	33	27	30
Total samples tested	42	24	18	16

Assay results				
Below limit of quantification (<10 μg/L)	31 (73.8)	18 (75.0)	19 (94.4)	15 (93.8)
Quantifiable; not protective (≥10 μg/L to <40 μg/L)	8 (19.1)	6 (25.0)	1 (5.6)	1 (6.2)
Quantifiable; protective (≥40 μg/L)	3 (7.1)	0 (0)	0 (0)	0 (0)

Self‐reported adherence				
Sub‐optimal adherence (<90% score)	9 (21.4)	3 (12.5)	3 (16.7)	4 (25.0)
Optimal adherence (≥90% score)	33 (78.6)	21 (87.5)	15 (83.3)	12 (75.0)

Abbreviation: PrEP, pre‐exposure prophylaxis.

## 4 DISCUSSION

In this cohort that studied oral PrEP preference, uptake, adherence and continuation, nearly half of AGYW preferred oral PrEP to other biomedical prevention methods, but uptake, adherence and PrEP continuation were low. Studies among men who have sex with men (MSM) show that 25–36% prefer oral PrEP to other options, including those in development, that is condoms, LA injectable PrEP and PrEP implants [16, 17]. A review of qualitative data from potential PrEP users, including AGYW, highlights that individual “preference” to use oral PrEP depends on how conveniently it can be incorporated into everyday life [18]. We show that AGYW preference for oral PrEP over other options is similar to other populations, and the low uptake, adherence and retention we observed indicate the need for more choices to meet individual needs.

Almost one third of volunteers started oral PrEP, which was lower than we expected among AGYW who expressed high willingness to use daily PrEP. Other studies also report low uptake among young people [10, 19], indicating that real‐world uptake has not reached clinical trial levels [20]. In a qualitative study done in family planning and youth clinics in Zimbabwe, where both oral PrEP and condoms were offered, PrEP uptake was low due to preference for condoms over PrEP [21]. Our assessment of oral PrEP preference was independent of condoms, which were also available; this likely affected PrEP uptake. Also, our volunteers were all <25 years, an age group that has continually shown lower PrEP uptake than older individuals [19, 22] likely due to their generally low HIV risk perception [23, 24]. In South Africa, mobile health clinics operating in locations frequented by AGYW are feasible, acceptable and improve PrEP uptake [25]. PrEP integration into sexual reproductive health services also facilitates uptake among AGYW [26]. These interventions could improve PrEP uptake among AGYW elsewhere in SSA.

Majority of volunteers started PrEP within 30 days of enrolment; however, two thirds discontinued PrEP, similar to the DREAMS implementation projects among AGYW in Kisumu and Homa Bay in Kenya [27]. The reasons we report for PrEP discontinuation have been reported among AGYW elsewhere [11, 28, 29]. Our site received monthly PrEP supplies from the regional PEPFAR Implementing Partner; we, therefore, gave volunteers monthly re‐fills, which was more frequent than anticipated as we preferred all activities to fall within the quarterly schedule. Frequent re‐fills could have led to fatigue and early disengagement in our cohort. However, this may only explain a limited number of drop outs, as a PEPFAR program implemented in fishing communities and trading centres in South‐Central Uganda gave less frequent (3‐monthly) re‐fills but also reported high discontinuation (median PrEP time, 45 days) [30]. Additionally, a trial that delivered a cognitive behavioural intervention among AGYW in Kampala reported that AGYW are highly mobile, which impacts access and retention to health services and research activities [31]. With emergence of Covid‐19, movement was restricted starting early 2020, public transport and several businesses were closed, while entertainment facilities that employed our volunteers remained closed until study end. This affected retention as we were unable to see volunteers and take samples. Adaptations to PrEP delivery were necessary. For example, the Sisters with a Voice (Sisters) program among female sex workers (FSWs) in Zimbabwe increased PrEP initiation rates by scaling up differentiated service delivery, giving multi‐month PrEP refills and using phone calls and WhatsApp for appointment scheduling, and post‐PrEP initiation support [32]. Similarly, we forecasted PrEP refills, received more stock and gave volunteers longer re‐fills through clinic and community visits.

Throughout the study, the proportion with detectable, protective blood TDF levels was very low and only in the first 3 months. In the HIV Prevention Trials Network (HPTN) 082 trial, only 20% of 16‐ to 25‐year‐old AGYW had high adherence, and detectable TDF levels declined from 84% (month 3) to 31% (month 12) [33]. Similar decreases in the proportion with detectable and protective drug levels over time have been observed among both young and adult MSM taking PrEP [34, 35]. We also show that TDF levels were lower than self‐reported adherence; a discrepancy reported in other studies [35, 36]. It may be difficult to achieve adequate adherence with protracted daily dosing; although volunteers reported high adherence, TDF assessments indicated that they were unable to take pills as prescribed. These findings support alternative oral products, such as LA pills, which will increase the options for AGYW who prefer the pill but not daily dosing [37]. Plasma TDF levels reflect recent dosing, and the low levels we report indicate that volunteers did not change their pill‐taking behaviour towards the clinic visits as reported in a study that assessed “white coat adherence” [38]. As PrEP rollout continues, assessment of samples for short‐term adherence, for example dry blood spots and urine, and long‐term adherence, for example hair samples, should take into account different challenges [39, 40]. These may continue to be assessed alongside self‐reported adherence, which is already widely used in routine care. Self‐reports can also be improved through Audio Computer‐Assisted Self‐Interviews, which allow volunteers to answer sensitive questions in private [41].

Preference for oral PrEP over other biomedical options was associated with 50% higher PrEP uptake. Gombe et al. have also shown that PrEP uptake in the public sector in Zimbabwe was driven by method preference [21]. Novel biomedical products will have similar modes of delivery to available contraceptives; lessons from contraceptive uptake can, therefore, be extended to HIV prevention. For example, low contraceptive uptake among women in SSA is driven by the absence of approved options of their preference [42, 43]. Development of HIV prevention products in consideration of available contraceptives gives the opportunity for multi‐purpose prevention technologies for prevention of HIV and pregnancy among AGYW.

PrEP uptake was higher among those with more sexual partners. Individual behavioural risks have similarly predicted PrEP uptake among AGYW and young black MSM [19, 44]. Furthermore, a higher proportion of volunteers with ≥10 sexual partners reported condom use in the 3 months before enrolment, indicating that they had high HIV risk perception, hence, readily started PrEP when it became available. Other studies among AGYW also show higher PrEP uptake with high self‐perceived HIV risk [19, 45]; this could facilitate uptake among volunteers who were separated as almost all reported paid sex. Messages should promote oral PrEP as a lifestyle choice that individuals can make during periods of risk.

### 4.1 Limitations

We used non‐random sampling, which is prone to selection bias that affects the generalizability of findings. Categorization of oral PrEP preference into a binary variable likely contributed to lower uptake, as it also included those who ranked it second. The Cockroft–Gault formula may overestimate glomerular filtration rate (GFR) among young people [46], the CKD‐EPI formula is, therefore, preferred. However, follow‐up creatinine and GFR evaluation remained within baseline ranges showing that PrEP use remained safe. Also, emancipated and mature minors may not be representative of all minors who are vulnerable to HIV, including those who need parental consent for health interventions. Despite the limitations, this study contributes to the growing literature on oral PrEP and highlights the importance of: end‐user preferences in intervention uptake, objective adherence assessments and community delivery during restricted movement.

## 5 CONCLUSIONS

Despite seemingly high levels of preference and acceptance, PrEP uptake and adherence were low; uptake was associated with higher sexual behavioural risk and oral PrEP preference. Development of alternative biomedical products should be expedited to meet end‐user preferences and community delivery promoted during restricted movement.

## COMPETING INTERESTS

The authors have no competing interests to declare.

## AUTHORS’ CONTRIBUTIONS

YM (lead author) contributed to study design, study coordination, data acquisition, analysis and interpretation, obtained funding, wrote the initial draft and revised versions of the manuscript. OK contributed to study design and carried out data analysis. JFL coordinated the data management. VMK contributed to study design. AB and DO performed tests for TDF levels in plasma. KC, MK and MAP contributed to study design. PK directed study implementation. All authors contributed to the interpretation of study results and critically commented on all versions of the manuscript. They approved the final version of the manuscript. All authors attest they meet the ICMJE criteria for authorship.

## FUNDING

This work was funded by IAVI and made possible by the support of many donors, including United States Agency for International Development (USAID) through the IAVI/ADVANCE program, grant number AID‐OAA‐A‐16‐00032. The full list of IAVI donors is available at http://www.iavi.org. IAVI also sponsored the study and therefore contributed to the study design, monitored the study and, reviewed and approved all versions of the manuscript.

## DISCLAIMER

The contents of this manuscript are the responsibility of the authors and do not necessarily reflect the views of USAID or the US Government.

## Supporting information


**Supplementary Table**: Table Showing Proportion of visits and Type of Visit during the Pre‐COVID Period, Lockdown and Post‐ Lockdown Periods.
**Supplementary Figure**: Comparison of Visits during the Pre‐COVID, COVID Lockdown and Post Lockdown Periods.

**Supplementary Table**: Monthly Self‐Reported Adherence Data of AGYW on PrEP.
**Supplementary Figure**: Bar graphs showing Quarterly Protective and Non‐Protective Plasma Tenofovir Levels of AGYW on PrEP.

## Data Availability

The datasets used and analysed during the current study are available from the corresponding author on reasonable request.
