# Supplementary material for: Oral pre‐exposure prophylaxis preference, uptake, adherence and continuation among adolescent girls and young women in Kampala, Uganda: a prospective cohort study
Source: J Int AIDS Soc. 2022 May 11;25(5):e25909. doi: 10.1002/jia2.25909 (PMC9092160; doi:10.1002/jia2.25909)
Supplement: Supplementary file 1 — Supplementary Table: Table Showing Proportion of visits and Type of Visit during the Pre‐COVID Period, Lockdown and Post‐ Lockdown Periods. Supplementary Figure: Comparison of Visits during the Pre‐COVID, COVID Lockdown and Post Lockdown Periods. [file JIA2-25-e25909-s001.docx]

**Effect of the COVID-19 Lockdown on Study Visits**

**Supplementary Table: Table Showing Proportion of visits and Type of Visit during the Pre-COVID Period, Lockdown and Post- Lockdown Periods.**

| **Period** | **Scheduled visits (N)** | **Attended on schedule (n/%)** | **Late Visits (n/%)** | **Missed Visits (n/%)** |
| --- | --- | --- | --- | --- |
| Pre-COVID (before 25^th^ March 2020) |  |  |  |  |
| Month 9 | 123 | 57 (46.3) | 10 (8.1) | 56 (45.6) |
| Month 12 | 80 | 33 (41.2) | 24 (30.0) | 23 (28.8) |
| Lockdown (25^th^ March – 31^st^ May 2020) |  |  |  |  |
| Month 9 | 79 | 16 (20.2) | 18 (22.8) | 45 (57.0) |
| Month 12 | 36 | 0 (0) | 22 (61.1) | 14 (38.9) |
| Post Lockdown (From 1^st^ June 2020 onwards) |  |  |  |  |
| Month 9 | 61 | 38 (62.3) | 6 (9.8) | 17 (27.9) |
| Month 12 | 147 | 71 (48.3) | 29 (19.7) | 47 (32.0) |

**Supplementary Figure: Comparison of Visits during the Pre-COVID, COVID Lockdown and Post Lockdown Periods.**
