# Supplementary material for: Oral pre‐exposure prophylaxis preference, uptake, adherence and continuation among adolescent girls and young women in Kampala, Uganda: a prospective cohort study
Source: J Int AIDS Soc. 2022 May 11;25(5):e25909. doi: 10.1002/jia2.25909 (PMC9092160; doi:10.1002/jia2.25909)
Supplement: Supplementary file 2 — Supplementary Table: Monthly Self‐Reported Adherence Data of AGYW on PrEP. Supplementary Figure: Bar graphs showing Quarterly Protective and Non‐Protective Plasma Tenofovir Levels of AGYW on PrEP. [file JIA2-25-e25909-s002.docx]

**Supplementary Table: Monthly Self-Reported Adherence Data of AGYW on PrEP.**

| **Month** | **Number Assessed** | **Optimal Adherence/**  **>=90**  **n (%)** |
| --- | --- | --- |
| M1 | 75 | 52 (69.4) |
| M2 | 56 | 48 (85.0) |
| M3 | 45 | 38 (85.0) |
| M4 | 35 | 30 (87.1) |
| M5 | 27 | 26 (95.5) |
| M6 | 33 | 33 (100) |
| M7 | 32 | 27 (85.0) |
| M8 | 31 | 29 (94.1) |
| M9 | 27 | 23 (85.7) |
| M10 | 31 | 29 (94.1) |
| M11 | 33 | 28 (84.6) |
| M12 | 30 | 28 (93.3) |


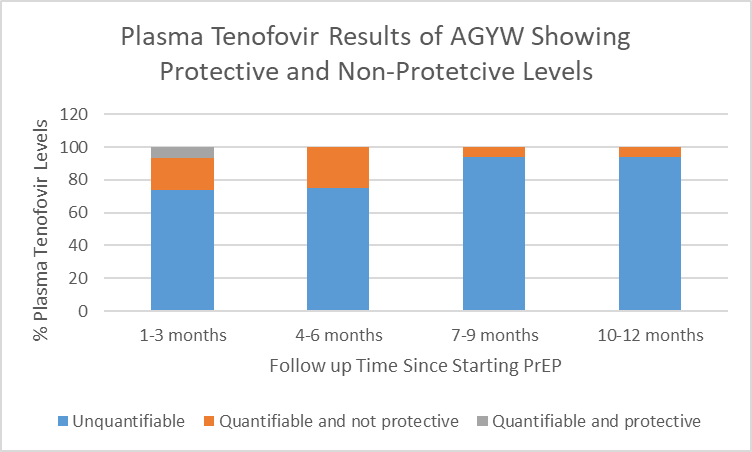


**Supplementary Figure: Bar graphs showing Quarterly Protective and Non-Protective Plasma Tenofovir Levels of AGYW on PrEP.**
